# Lactate activates hypothalamic POMC neurons by intercellular signaling

**DOI:** 10.1038/s41598-021-00947-7

**Published:** 2021-11-04

**Authors:** P. Órdenes, P. S. Villar, E. Tarifeño-Saldivia, M. Salgado, R. Elizondo-Vega, Ricardo C. Araneda, María A. García-Robles

**Affiliations:** 1 Laboratorio de Biología Celular, Departamento de Biología Celular, Facultad de Ciencias Biológicas, Universidad de Concepción, Concepción, Chile; 2 Department of Biology Bioscience Research Bldg R-1114, University of Maryland, College Park, MD 20742 USA; 3 Gene Expression and Regulation Laboratory, Departamento de Bioquímica y Biología Molecular, Facultad de Ciencias Biológicas, Universidad de Concepción, Concepción, Chile

**Keywords:** Cell biology, Neuroscience

## Abstract

Previous studies indicate that the activity of hypothalamic POMC neurons can be regulated by glucose via intracellular mechanisms, but its regulation by lactate is poorly understood. In addition to its energetic role, lactate acts as a signaling molecule. In this study, we evaluated the function and location of the lactate receptor, hydroxycarboxylic acid receptor 1 (HCAR1). We used a conditional genetic approach to label POMC neurons and evaluated their sensitivity to lactate using patch-clamp recordings. l-Lactate and 3-chloro-5-hydroxybenzoic acid (3Cl-HBA), HCAR1 specific agonist depolarized POMC neurons and the increase in excitability was abolished by pertussis toxin (PTX), indicating the involvement of Gαi/o-protein-coupled receptors. In addition, the depolarization of a subset of POMC neurons was sensitive to α-cyano-4-hydroxycinnamate (4-CIN), a lactate transporter blocker, suggesting that the depolarization induced by l-lactate can also occur by direct intracellular action. Surprisingly, HCAR1 was not detected in POMC neurons, but instead localized in astrocytes. These results suggest a new lactate-mediated mechanism for astrocyte-neuron intercellular communication.

## Introduction

The arcuate nucleus (ARC), located in the basal hypothalamus, contains neurons that integrate peripheral signals of the body energy status and regulate feeding behavior and glucose homeostasis^1–3^. Detection of variations in extracellular glucose concentrations is an important mechanism in the neuroendocrine regulation of feeding behavior by the hypothalamus. Glucose-sensitive neurons are mainly found in the ARC and ventromedial nucleus (VMN) of the hypothalamus^4–9^. Several types of glucosensing neurons have been characterized according to their response to changes in extracellular glucose concentration. Whereas glucose-excited (GE) neurons increase their firing as extracellular glucose concentration increases, glucose-inhibited neurons (GI) decrease their firing under similar conditions^4,7^. In GE neurons, responses to glucose may be mediated by the closure of ATP-sensitive K^+^ channels (K_ATP_), while in GI neurons, glucose induces the opening of Cl^−^ channels^5,7–10^. Furthermore, the hypothalamic melanocortin pathway plays a critical role in synthesizing orexigenic and anorexigenic and neuropeptides, which are regulated by hormones and nutrients^11–13^. First, orexigenic neurons synthesize the neuropeptides, NPY and AgRP, which increase appetite and decrease energy expenditure, and these neurons have been shown to correspond to GI neurons^4,14–16^. In contrast, POMC neurons synthesize the anorexigenic neuropeptide, α-MSH, which is derived from the proopiomelanocortin transcript^17–19^, inducing satiety and increasing energy expenditure^20–22^. Electrophysiological studies have shown that approximately 50% of POMC neurons are GE^6,10^, while others have reported that ~ 95% of POMC neurons do not respond to glucose^4,9^.

In addition to intrinsic responses to glucose, POMC neurons can be regulated by molecules released from nearby glia cells. For example, tanycytes, a type of glia that cover the basal walls of the third ventricle (3 V), release lactate into ARC neurons^23^, which could also be incorporated into POMC neurons via the monocarboxylate transporter, MCT2^24^, resulting in depolarization through the closure of K_ATP_^8^. Additionally, POMC neurons could receive synaptic input from other glucose-sensitive neurons^25^. However, there is no consensus on the mechanism underlying the modulation of POMC neuron electrical activity by glucose or its metabolites. Here, we use a conditional genetic approach to selectively label POMC neurons in the ARC and conduct targeted electrophysiological recordings to assess their responses to l-lactate and related agonists. We show that most POMC neurons exhibit an excitatory response to lactate mediated by the activation of the lactate receptor, HCAR1. In a minor subpopulation of neurons, the response to l-lactate appears to be mediated via intracellular transport. Interestingly, HCAR1 immunoreaction was absent in POMC neurons, but was detected in astrocytes. Taken together, these data represent a significant advance in the understanding of the role of lactate in regulating the activity of POMC neurons.

## Results

### Hypothalamic POMC neurons respond to l-lactate

**Figure 1:**
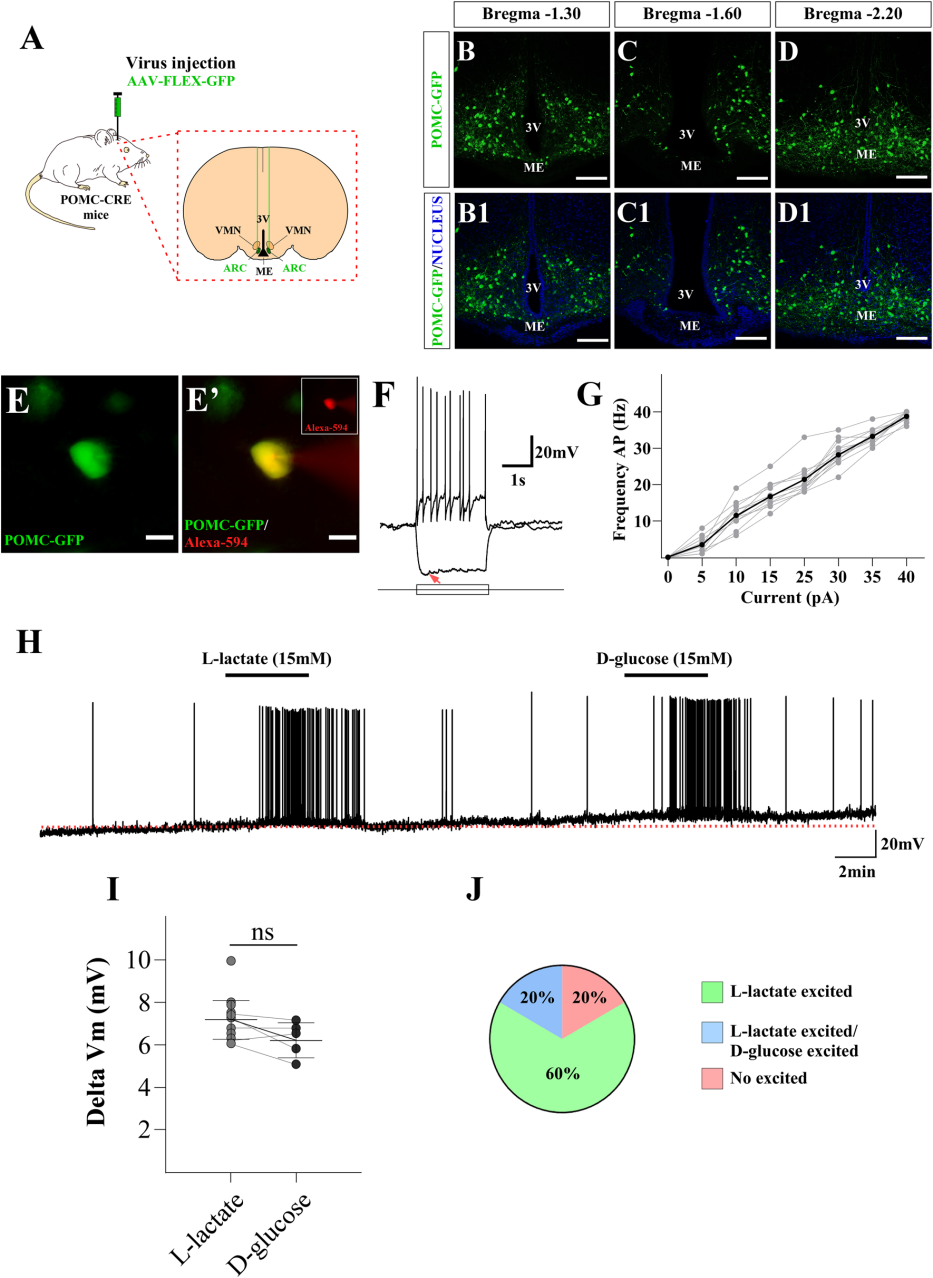
Lactate-excited POMC neurons in the ARC. (**A**) Scheme illustrating the experimental approach. To target the expression of GFP specifically in POMC neurons, we bilaterally injected AAV-Flex-GFP into the ARC of POMC-Cre mice. (**B–D1**) 14 days there was abundant antero-posterior distribution of GFP-labeled POMC neurons in the ARC (scale bar 150 µm). (**E**) Low magnification of POMC-EGFP neurons in the ARC (green); (**E´**) Same neuron (yellow) after dialysis of the Alexa-594 dye (red) through the recording pipette. (**F**) Responses in current clamp to stimulation with negative and positive square current pulses (scale bar 20 µm) (red arrow: current activated by hyperpolarization). The resting membrane potential is − 58 mV in this cell. (**G**) Input–output response to increasing current injection; under these conditions POMC neurons fired up to at least 40 ± 1 Hz. (**H**) Response of POMC neuron to 15 mM l-lactate and 15 mM d-glucose; both metabolites produced a small depolarization accompanied by an increase in firing. The resting membrane potential in this neuron was − 55 mV. (**I**) At the tested concentration, both metabolites produce a similar change in membrane potential of POMC neurons. (**J**) Graph illustrating the percentage of POMC neurons that respond to l-lactate and d-glucose. Baselines are shown with the red dotted lines.

To characterize the responses to glucose and lactate in POMC neurons, we conducted targeted current-clamp recordings in GFP-expressing neurons. POMC neurons were labeled by bilateral injections of a Cre-dependent AAV-FLEX-GFP virus in the ARC of POMC-Cre mice, a transgenic line commonly used to assess the function of POMC neurons^26–30^ (Fig. 1A). In agreement with previous work using different viruses and reporter^28,31^, 14 days post-injection we detected abundant expression of GFP in POMC neurons, across the entire anteroposterior axis of the ARC (Fig. 1B–D1). To further confirm the identity of the recorded GFP-labeled neurons, we included the red fluorophore, Alexa-Fluor 594, in the internal solution (Fig. 1E-E’). The mean resting membrane potential of POMC neurons was − 55 ± 2 mV, under zero current injection, and the mean input resistance was 1.36 ± 0.40 GΩ (n = 12). Stimulation of POMC neurons with current steps of increasing amplitude resulted in trains of action potentials that reached a maximal firing rate of 40 ± 1 Hz (Fig. 1F,G), while negative current steps elicited a sag in the membrane potential consistent with the presence of the hyperpolarization activated current, I_h_ (6/12) (Fig. 1F, redarrow). These properties of POMC neurons are similar to those described previously using the POMC-EGFP mouse^6^. To evaluate the responses of POMC neurons to l-lactate, we used a concentration of 15 mM^32^, since basal plasma concentrations of lactate are in the range of 0.5 and 2 mM, but under certain conditions, such as intensive exercise, plasma levels are as high as 20 mM similar to plasma glucose concentration after food intake^33–35^. As shown in Fig. 1H,I, bath perfusion of 15 mM l-lactate reversibly depolarized POMC neurons (Fig. 1H,I ∆Vm 7.37 ± 0.75 mV n = 20). Moreover, a subset of POMC neurons excited by l-lactate (5/25) also depolarized in response to the same concentration of d-glucose (Fig. 1H,I; ∆Vm 6.58 ± 0.68 mV). Similar results were obtained using the POMC-EGFP mice model (Supplementary Fig. S2). In Fig. 1J, we summarize the responses to lactate and glucose in the total population of POMC neurons recorded. Out of 25 recorded neurons, 15 were activated only by l-lactate (60%), while five were activated by both glucose and lactate (20%). Lastly, five of the recorded neurons were unresponsive to either l-lactate or d-glucose (20%). These results suggest that excitatory responses to l-lactate in POMC neurons are prevalent.

### Responses to l-lactate in POMC neurons are heterogenous

**Figure 2:**
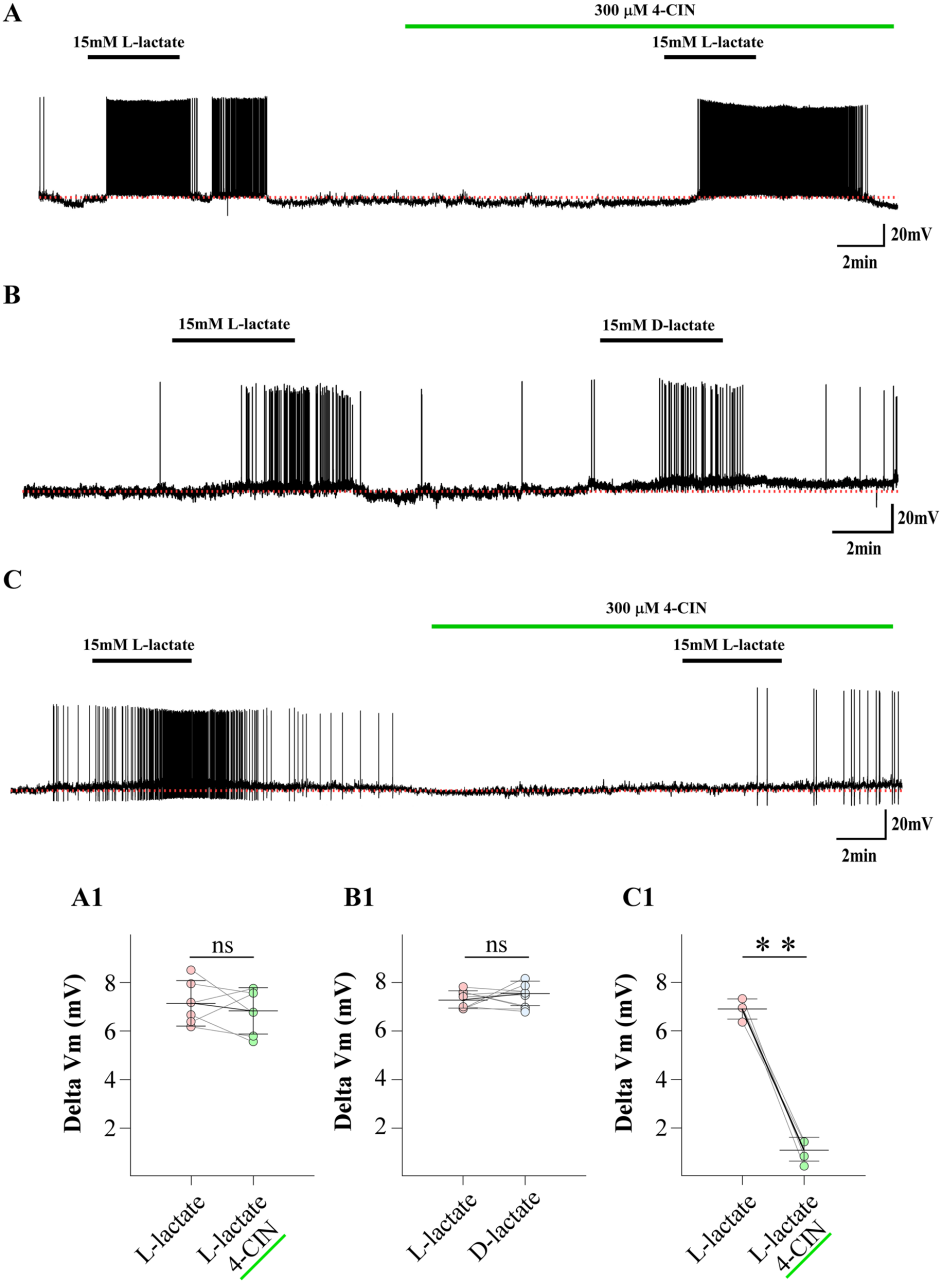
The excitatory effect of l-lactate in POMC neurons depends only partially on lactate uptake. (**A**) The depolarization elicited by l-lactate was not abolished by the MCT inhibitor (300 µM 4-CIN). The resting membrane potential was − 55 mV. (**B**) Example of a POMC neuron excited by l-lactate (15 mM) that was also depolarized by the non-transportable isomer, d-lactate (15 mM). The resting membrane potential was − 54 mV. (**C**) The depolarization elicited by l-lactate was abolished by the MCT inhibitor (300 µM 4-CIN). The resting membrane potential was − 56 mV. (**A1–C1**) Summary of the effects of l-lactate, 4-CIN/l-lactate and d-lactate on the excitability of POMC neurons. Baselines are shown with the red dotted lines. Results represent the mean ± SD of ten different cells for recordings. **p < 0.01 one tailed test.

l-Lactate is incorporated in hypothalamic neurons mainly by the MCT isoform 2 (MCT2) via a H^+^-mediated symport mechanism^24^, while the stereoisomer d-lactate is not transported by MCTs^36,37^. Therefore, we examined whether the depolarization of POMC neurons elicited by l-lactate could be affected by the presence of 4-CIN, a general MCT inhibitor^38^. The half-maximal inhibitory concentration of 4-CIN to reduce l-lactate transport through MCT2 by 4-CIN is 24 μM^39^; therefore, we used a concentration of 300 μM to ensure total inhibition. The l-lactate response was not significantly affected by 4-CIN (60%); the ∆Vm for l-lactate was 7.18 ± 0.95 mV vs 6.87 ± 0.97 mV for l-lactate/4-CIN (Fig. 2A,A1). Interestingly, we found that most POMC neurons excited by 15 mM l-lactate (nine of 10) were also depolarized by a similar magnitude in the presence of 15 mM d-lactate (Fig. 2B,B1; ∆Vm, l-lactate, 7.36 ± 0.49 mV; d-lactate, 7.37 ± 0.51 mV). In the remaining neurons (40%), 4-CIN significantly reduced the depolarization produced by 15 mM l-lactate (Fig. 2C,C1; ∆Vm, l-lactate, 6.95 ± 0.41 mV vs l-lactate/4-CIN, 1.0 ± 0.49 mV). These data suggest that over half of the responses to l-lactate in POMC neurons occur via a mechanism that is independent of its transport, which is the focus of the sections.

### HCAR1 is functionally expressed in the ARC

**Figure 3:**
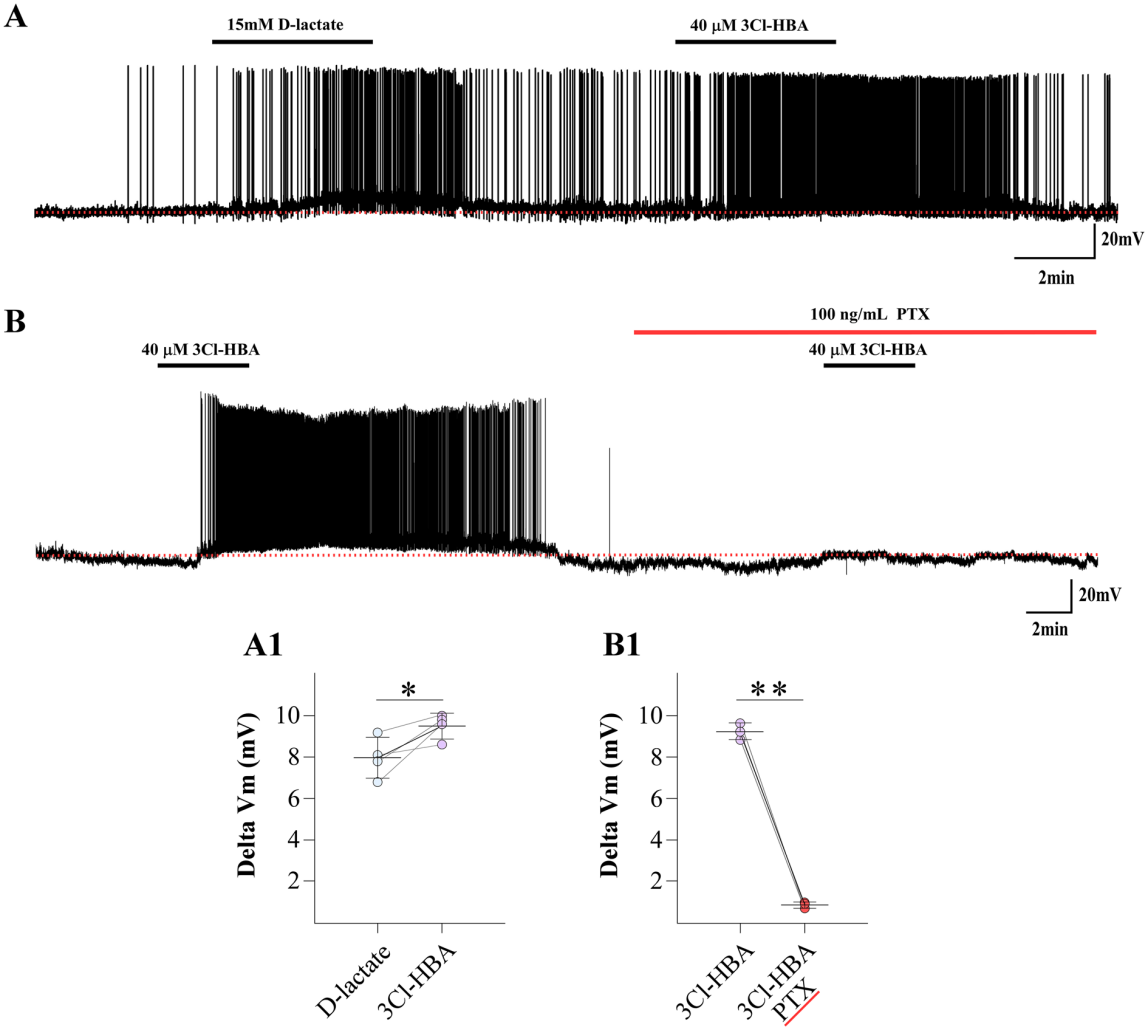
Activation of HCAR1 depolarizes POMC neurons. (**A**) POMC neurons excited by d-lactate (15 mM) were also depolarized in the presence of the HCAR1 agonist, 3Cl-HBA (40 µM). The resting membrane potential was − 54 mV. (**B**) The depolarization elicited by 3Cl-HBA was completely abolished by the Gαi protein inactivator (100 ng/mL PTX). The resting membrane potential was − 56 mV. (**A1–B1**) Summary of the effects of d-lactate/3-Cl HBA and 3-Cl HBA/PTX on the membrane potential in POMC neurons. Baselines are shown with the red dotted lines.

Previous studies have determined that d-lactate is a partial HCAR1 agonist^40,41^; thus, we hypothesized that the excitatory effect of d-lactate is mediated by activation of HCAR1. However, whether HCAR1 is expressed and functional in the ARC is unknown. Thus, we examined the responses of POMC neurons to d-lactate (15 mM) and 3Cl-HBA (40 µM), a specific HCAR1 agonist^42,43^. Interestingly, we found that POMC neurons excited by d-lactate exhibited a robust depolarization in the presence of 3Cl-HBA (Fig. 3A,A1; ∆Vm, d-lactate, 7.98 ± 0.98 mV; 3Cl-HBA, 9.50 ± 0.62 mV; n = 4). Furthermore, HCAR1 is a Gi/o-protein-coupled receptor^40,41,44^, therefore, we examined the ability of 100 ng/mL pertussis toxin, (PTX), a Gi protein inhibitor^41,45,46^, to reduce the depolarizing responses. As shown in Fig. 3B, POMC neurons pre-incubated with PTX showed a significant reduction in their response to 3Cl-HBA (Fig. 3B1; ∆Vm, 3Cl-HBA, 8.7 ± 0.40 mV vs 3Cl-HBA/PTX, 0.87 ± 0.15 mV, n = 3).

### HCAR1 is expressed in the hypothalamus, but not in POMC neurons

HCAR1 is a Gi-coupled receptor, and its activation decreases calcium waves and excitability in neurons^36,42^. To determine how the activation of this inhibitory receptor could depolarize POMC neurons, we analyzed the localization of HCAR1 in the ARC of POMC-EGFP transgenic mice using immunohistochemical analyses. As a first step, we determined the expression of HCAR1 in the hypothalamus and control tissues using qRT-PCR and Western blot (Supplementary Fig. S1A–C). In agreement with previous reports^47–49^, HCAR1 was detected in the cerebellum and in the cortex, where it was localized in neuronal-like type cells. In contrast, HCAR1 was detected mainly in astrocytes of the hippocampus (Supplementary Fig. S1D–G, blue arrows).

**Figure 4:**
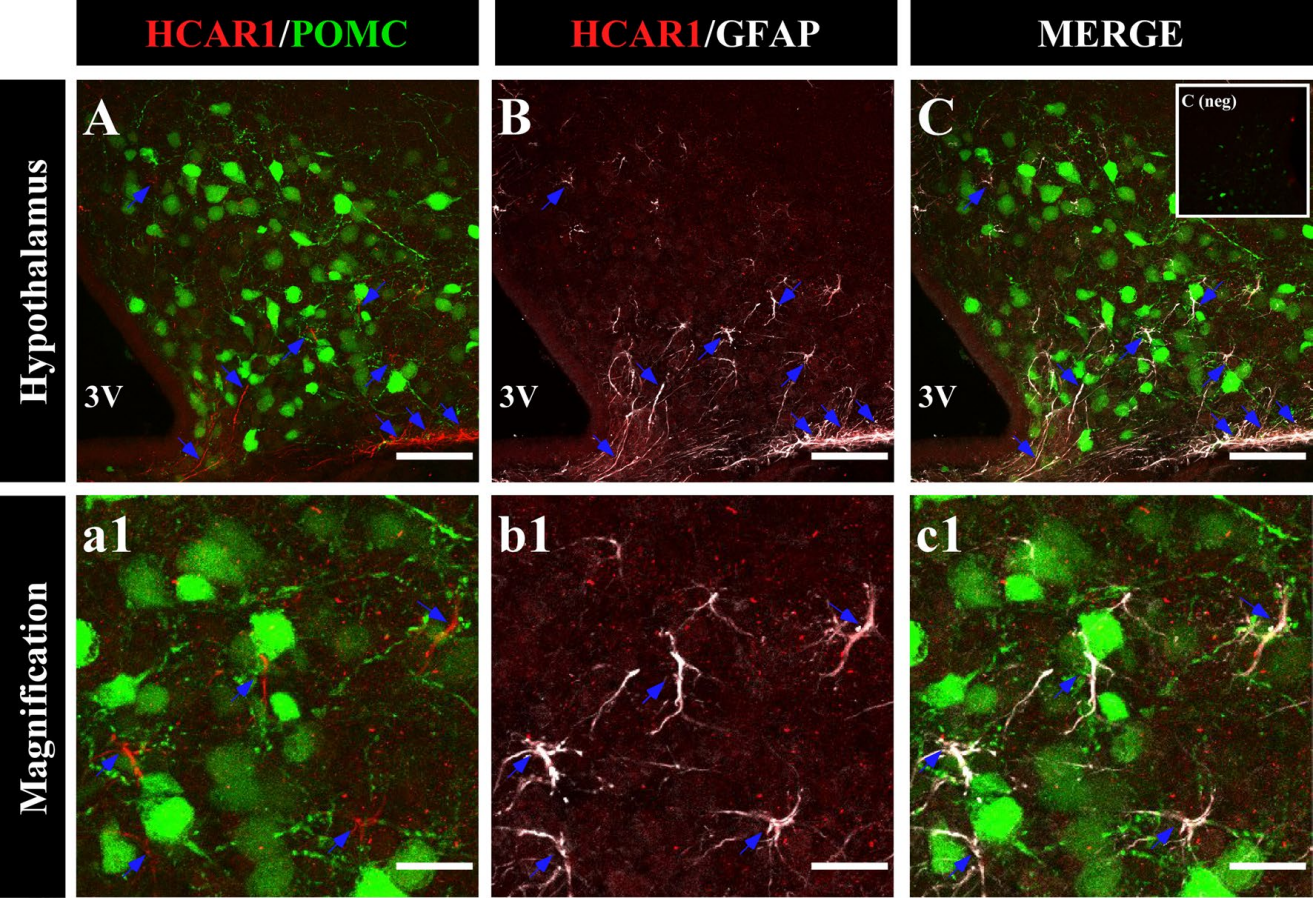
HCAR1 is localized in hypothalamic astrocytes. (**A–C**) Coronal sections of POMC-EGFP transgenic mice were immunolabeled with HCAR1 antibody (red). The same slices were revealed using GFAP antibody (white), an astrocyte marker. HCAR1 and GFAP were distributed in ARC astrocytes (**a1–c1**), high magnification images showed astrocytes localized in the proximity of POMC neurons blue arrow (**a1**–**c1**). Scale bar (**A**–**C**) 100 µm. (**a1**–**c**1) 50 µm.

**Figure 5:**
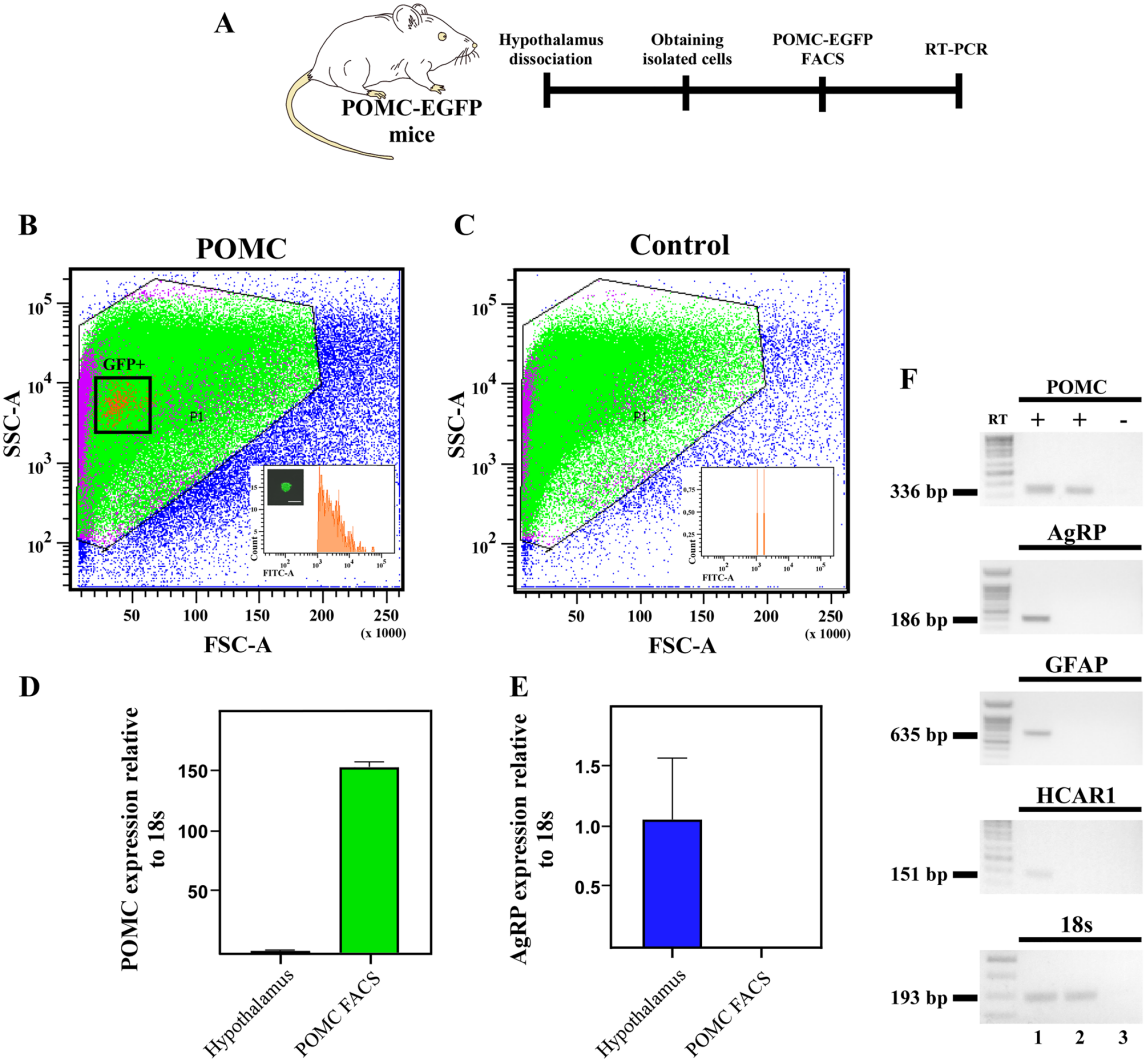
HCAR1 is absent of POMC neurons. (**A**) Experimental workflow. (**B**,**C**) Size versus cellular complexity plots from FACS purification of POMC-EGFP neurons from transgenic mice. Inserts correspond to FITC intensity distribution of sorted events. (**B**) POMC-EGFP mice. (**C**) WT control mouse. (**D**,**E**) qRT-PCR of hypothalamus samples and sorted POMC neurons in POMC-GFP mice (N = 3). (**F**) Expression profiles of Pomc, Agrp, Gfap, Hcar1 and 18 s ribosomal RNA from hypothalamus samples (lane 1), sorted POMC neurons (lane 2) cDNA, RT (−) of sorted POMC neurons (lane 3).

Surprisingly, despite the robust excitatory responses in POMC neurons in the presence of HCAR1 activation, confocal imaging of hypothalamic sections from the POMC-EGFP mice, failed to show immunoreactivity for HCAR1 in POMC neurons (Fig. 4A). HCAR1 immunoreactivity in the ARC was detected mainly in astrocyte-like cells, and HCA1R co-localized with GFAP, an astrocyte marker (Fig. 4B,C, blue arrows). Higher magnification, revealed the presence of HCAR1 distributed in astrocytes in close apposition to POMC neurons (Fig. 4a1–c1). To confirm the absence of the receptor in POMC neurons, EGFP-POMC neurons from transgenic mice were purified by FACS (Fig. 5A). Hierarchical sorting profiles are shown in Fig. 5B,C; inner plots display the fluorescent intensity of sorted events, indicating high enrichment of GPF-positive cells in the purified neurons. Of note, GFP-positive cells constitute a morphologically homogenous population in terms of cell size and complexity as shown in Fig. 5B. We successfully isolated 497 GFP-positive events from four POMC-EGFP transgenic mice. To evaluate the enrichment of POMC neurons in sorted cells, we compared the hypothalamic Pomc mRNA with POMC neurons obtained by FACS. For this, mRNA samples obtained from the hypothalamus and sorted GFP-positive cells were subjected to Pomc and Agrp qRT-PCR and normalized to 18S ribosomal RNA and hypothalamic samples (Fig. 5D,E). The level of Pomc mRNA expression was 150 times greater in sorted cells than in hypothalamic samples (Fig. 5D). In contrast, and as expected, Agrp mRNA expression was absent in the sorted POMC cells (Fig. 5E). Subsequently, we analyzed tissue samples by conventional PCR. Consistent with the immunolocalization results, hypothalamic tissue expressed Hcar1, while no signal was detected in the sorted samples (Fig. 5F). In addition, Gfap, Pomc, and Agrp mRNA was detected in total hypothalamic samples, however, only Pomc expression was detected in EGFP-sorted cells (Fig. 5F, line 2). Collectively, these data suggest that the increase in POMC neuron excitability elicited by l-lactate is mediated by activation of HCAR1 located in astrocytes.

## Discussion

POMC neurons are present in the ARC, and their activation induces α-MSH release, which acts over MCR4 receptors located in different brain areas for increasing energy expenditure and reducing food intake^21^. POMC neurons are activated by nutrient availability and leptin among others. Contradictory evidence suggests that glucose activates POMC neurons through K_ATP_^6,10^ while other studies showed that a low number of POMC neurons are glucose-responsive^4,9^. Our electrophysiological analysis demonstrated that POMC neurons are activated by l-lactate, which is consistent with a behavioral study that demonstrated intracerebroventricular (icv) l-lactate injection reduces food intake^50^. In this context, glial cells are eminently glycolytic and release lactate in vitro^23^. Therefore, lactate originating from glial cells could activate POMC neurons^23,24^.

To the best of our knowledge, this is the first study showing the expression and function of HCAR1 in the ARC and that l-lactate depolarized POMC neurons via the activation of this receptor. HCAR1 was first described in adipocytes^51^, and later studies described its expression in the cerebellum, hippocampus, and brain cortex^47–49^. Functional studies have indicated that this receptor is coupled to Gi/o^40,42,45,48,51^, and activation is associated with reduced excitability in neurons^52^. Similarly, in cortical neurons, activation of HCAR1 by lactate decreases neuronal excitability^36,42^. However, an electrophysiological study carried out in acute slices of the hippocampus showed l-lactate and a specific agonist of HCAR1 increased neuronal firing rate^45^. In a similar way, our data showed that l-lactate, d-lactate, and 3Cl-HBA depolarized POMC neurons, suggesting a non-canonical mechanism of POMC neuron activation by lactate.

Our immunohistochemical studies showed that HCAR1 is localized in astrocytes of the ARC. Similar cellular expression was reported in hippocampal astrocytes^48^. To the best of our knowledge, there are no functional studies on the modulation of HCAR1 excitability in astrocytes. However, an elegant analysis using DREADDs technology combined with electrophysiological techniques analyzed the effect of the exogenous expression of hM4Di (a GPCR-Gi) in hippocampal astrocytes over neuronal excitability^52^. The authors demonstrated that the hM4Di activation with CNO increased intracellular calcium in astrocytes lead to glutamate release and depolarization of neighboring neurons^52^. Therefore, it is plausible to propose that POMC depolarization occurs through a similar mechanism. Notably, using a similar strategy to express hM4Di in hypothalamic astrocytes, the authors reported that the CNO treatment increased the firing rate of POMC neurons^53^.

**Figure 6:**
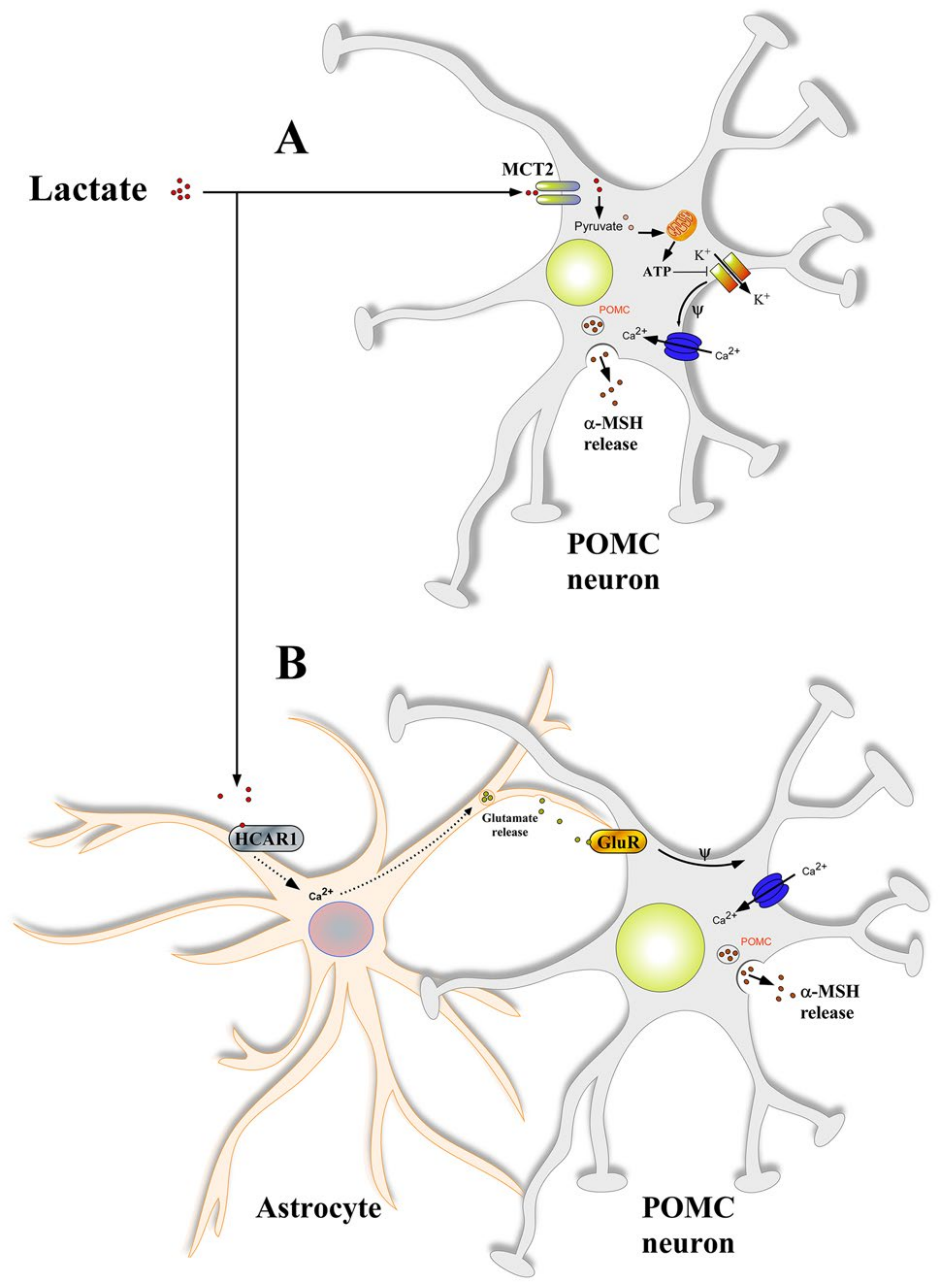
Mechanisms proposed for lactate to depolarize POMC neurons of the hypothalamus. In a high-energy condition, lactate modulates the activity of POMC neurons through two pathways: (1) directly through incorporation via MCT2 or indirectly by binding to HCAR1 in astrocytes. (**A**) In a classical mechanism of neuronal activation, lactate is taken up by neurons using MCT2 and serves as a suitable energetic substrate. The increase in the ATP/ADP ratio causes the closure of ATP-sensitive K^+^ channels, producing a depolarization. (**B**) The binding of lactate to HCAR1 in astrocytes activates a Gi/o-coupled pathway that, through an increase in intracellular calcium, leads to the release of glutamate onto POMC neurons. In this model, the activation of glutamate receptors will cause depolarization of POMC neurons. Dotted line shows a possible mechanism that has not been evaluated yet.

On the other hand, we showed that 40% of the POMC neurons recorded were excited by l-lactate by MCT dependent-mechanism, in agreement with results recently reported by Lhomme et al., using the same animal model (POMC-Cre)^54^. These results suggest that lactate could be incorporated and metabolized to produce ATP and use the intracellular mechanism of neuronal activation. Our electrophysiological study using D-glucose and l-lactate showed that POMC neurons respond primarily to l-lactate rather than D-glucose. The ability of lactate to supply glucose function was reported in physiological studies where l-lactate perfused in VMH in vivo suppresses the counterregulatory response to hypoglycemia^50^. Previous reports investigating the role of lactate in low-glucose conditions have demonstrated that 15 mM lactate increases the firing rate of VMH^32^. These studies lead to the hypothesis that lactate could play a role in inducing satiety, and our data support this hypothesis that l-lactate, produced by ventricular glia (i.e. tanycytes), increased the activity of POMC neurons. Consistent with this, our previous work in MCT knockdown-rats showed that tanycyte expression of MCT1 and MCT4 is required to maintain the response of POMC neurons to icv glucose and to normal food intake^55,56^. Importantly, microstructure analyses demonstrated that this effect was due to a decrease in the induction of satiety^55^. Similar results were recently obtained by Lhomme et al. in which electrophysiological recordings showed that POMC neurons are activated by glucose, a response that was lost with tanycytic MCT1-4 inhibition^54^. Additionally, they showed that inhibition of connexin 43 in tanycytes affected the excitability of POMC neurons and increased food consumption^54^. Similarly, we have demonstrated that tanycytes are not only coupled with each other but also with astrocytes and oligodendrocytes^57^, highlighting the important role of the glial cells in these processes. Our current results suggest a new lactate-mediated mechanism for glial-neuron intercellular communication. We propose that lactate can act through two mechanisms that result in the depolarization of POMC neurons: (1) a metabolic mechanism involving lactate incorporation, ATP production, closure of K_ATP_ channels, and α-MSH release and (2) a signaling mechanism where lactate-mediated activation of HCAR1 in astrocytes could lead to glutamate release and subsequent activation of POMC neurons (Fig. 6). More evidence is required to determine how lactate exerts its effects on neurons through glial cells and test whether HCAR1 activation in astroglial cells releases glutamate, which then activates POMC neurons. Therefore, our results stand out as a new lactate-mediated mechanism for glia-neuron intercellular communication.

## Materials and methods

### Animals

Animal experiments were performed in compliance with the ARRIVE guidelines (https://arriveguidelines.org). All studies performed at the University of Maryland were approved by the Institutional Animal Care and Use Committee (IACUC), following NIH guidelines. Mice were group-housed (2–4 siblings) at 22–24 °C with a 12-h light–dark cycle, and with ad libitum access to a regular chow diet and water. All diets were provided as pellets. POMC-Cre (Jax, 010714, RRID: IMSR_JAX:010714) mice were available at the Jackson Laboratory. We used this animal model in electrophysiological recordings (injected with AAV-FLEX-GFP), as this is transgenic line that has been used to evaluate the function of POMC neurons^26–30^. Studies performed at the Universidad de Concepción were approved and reviewed by the ethics committee of the Faculty of Biological Sciences and by the committee on Ethics, Care and Use of Animals of the Universidad de Concepción. Experimental procedures were performed using wild-type C57BL/6J mice (RRID: IMSR_JAX:00066) and C57BL/6J-Tg (POMC-EGFP) 1Low/J mice (RRID:IMSR_JAX:009593). Animals were arbitrarily assigned to experimental groups; no randomization was performed. All relevant information is provided in the manuscript and custom-made materials will be provided upon request.

### Stereotaxic surgery

Stereotaxic surgery to deliver AAV-FLEX-GFP (virus titer 2.3 × 10^13^) (Addgene Cat#28304, Watertown, MA) into the hypothalamus of mice was performed as previously described^28^. In brief, 12–16-week-old mice were anesthetized with isoflurane (1 mg/mL) delivered by veterinary vaporizer (Matrx VIP 300, Midmark, OH) and fixed on a stereotaxic apparatus (KOPF model Cat#940, Tujunga, CA) with auditory bars that preserve the tympanic membrane^58^. After exposing the skull via a small incision, small holes were drilled for injection based on coordinates to bregma (in mm): − 1.70 A-P, − 5.80 D-V, ± 0.30 M-L). A fine pulled-glass pipette was inserted into the brain, and AAV (80 nL per ARC) was injected by an in-house-built air-puff system. For postoperative care, mice were injected intraperitoneally with meloxicam (0.5 mg/kg) for two continuous days. All stereotaxic injection sites were verified under microscopy. After surgery, animals were allowed to recover for at least 1 week, and their body weight and health conditions were closely monitored during recovery.

### Slice preparation

Experiments were performed in hypothalamic slices obtained from 12 to 16-week-old POMC-Cre mice 14 days post virus injection, as previously done^59,60^. Briefly, animals were deeply anesthetized with isoflurane and decapitated. Brain slices were quickly prepared in a modified artificial cerebrospinal fluid (ACSF) having lower calcium (0.5 mM) and higher magnesium (3 mM) compared to normal ACSF. In all experiments, unless otherwise stated, the extracellular solution is ACSF had the following composition to give a pH 7.4 and an osmolarity of ~ 305 mOs: 125 mM NaCl, 25 NaHCO_3_, 1.25 mM NaH_2_PO_4_, 3 mM KCl, 2 mM CaCl_2_, 1 mM MgCl_2_, 3 mM myo-inositol, 0.3 mM ascorbic acid, 2 mM Na-pyruvate, and 1 mM d-glucose, continuously oxygenated (95% O_2_–5% CO_2_). Coronal slices of 250 μm thickness, containing the ARC were obtained with a vibratome Leica (VT 1200S; Leica Biosystems, Buffalo Grove, IL). Slices were then transferred to an incubation chamber containing normal ACSF and left to recuperate first at 35 °C for 30 min and then at room temperature until use.

### Whole-cell recordings and data acquisition

Slices were placed in a submerged recording chamber mounted on the stage of an upright microscope (BX51W1 DIC, Olympus, Center Valley, PA), fitted with differential infrared interference contrast (IR-DIC) optics, and perfused with 2 mL/min of ACSF at room temperature^59^. Current clamp recordings were performed using a dual EPC10 amplifier in current-clamp mode (HEKA, Union City, NY). Recording electrodes were made from borosilicate glass tubes (Sutter Instruments, Novato, CA) using a Flaming-Brown puller (Sutter Instruments, Novato, CA), and were filled with an internal solution of the following composition: 10 mM Na-Gluconate, 4 mM NaCl, 120 mM K-gluconate, 10 mM HEPES-K, 10 mM Na phosphocreatine, 2 mM Na-ATP, 4 mM Mg-ATP, and 0.3 mM GTP adjusted to pH 7.3 with KOH^59^. The osmolarity of the internal solutions was adjusted to 290–305 mOsM. The electrode resistance varied between 6 and 8 MΩ. Slices were observed with a 40X water immersion objective and visualized using a camera (CoolSNAP-EZ, Photometrics, Tucson, AZ). EGFP-fluorescence was detected using epifluorescence illumination. Data analysis was performed using macros written for the Igor Pro-software version 6.37 (Wavemetrics, Oswego, OR: www.wavemetrics.com)^59^. All values reported correspond to results from at least three experiments, and error bars indicate the SE. Statistical differences were assessed by the paired t test.

### Solutions and pharmacological agents

The following drugs were bath applied: d-( +) lucose ≥ 99.5% (Cat# G8270); Sodium l-lactate (Cat# L7022), Sodium D lactate (Cat# 71716), α-Cyano-4-hydroxycinnamate (Cat# C2020), 3-Chloro-5 hydroxybenzoic acid (Cat# 0447) from Sigma-Aldrich, St. Louis, MO, USA and Pertussis Toxin (Millipore-Sigma, Cat# 516560). The agonists were bath perfused for at least 3 min, and antagonists were applied for at least 10 min before the agonist. The speed of perfusion allowed for full solution exchange within 2 min.

### Quantitative reverse transcription-polymerase chain reaction (qRT-PCR)

Total RNA from hypothalamus, brain cortex, hippocampus, and cerebellum samples were isolated using TRIzol (Invitrogen Rockville, MD, USA) and treated with DNase I (Fermentas International, Burlington, Ontario, Canada). RT-PCR was performed according to the manufacturer’s protocol (Fermentas International) using 2 µg of RNA. Parallel reactions were performed in the absence of reverse transcriptase to control for the presence of genomic DNA. qRT PCR reactions were prepared with a Brilliant II SYBR Green qPCR Master Mix kit (Agilent Technologies, Santa Clara, CA, USA) in a final volume of 20 µL containing 2 µL cDNA and the following sets of primers (500 nM each): Hcar1, sense; 5′-ATC CTG GTC TTC GTG CTT GG-3′ and antisense 5′-CTG TCC GAA GGG GTA AGC AG-3′; Pomc sense 5′ TGA ACA GCC CCT GAC TGA AAA C 3′ and antisense 5′ AGG ACC TGC TCC AAG CCT AAT G 3′; Agrp sense 5′ GCA GAC CGA GCA GAA GAT GTT C 3′ and antisense 5′ GTA GCA CGT CTT GAA GAA GCG G 3′; Gfap sense 5′ CTC AAT GCT GGC TTC AAG GAG A 3′ and antisense 5′ GAC GCA GCG TCT GTG AGG TC 3′ and 18S ribosomal RNA sense 5′ GCC CGA AGC GTT TAC TTT GA and antisense 5′ TTG CGC CGG TCC AAG AAT TT 3′.

Each reaction mixture was incubated at 95 °C for 5 min followed by 40 cycles of 15 s at 95 °C, 15 s at 55 °C, and 15 s at 72 °C and a final extension of 7 min at 72 °C. The relative expression was calculated by the comparative 2^−ΔΔCt^ method using 18S ribosomal RNA as the housekeeping control gene^61^.

### Immunohistochemistry

Sections of the cerebellum, hippocampus, and frontal hypothalamus (40 µm) were fixed directly by vascular perfusion and post immersion fixation in 4% (w/v) paraformaldehyde for 24 h, and subsequently cut by vibratome and processing by free-floating. Tissues were stained with mouse anti-GFAP (1:200; Millipore Cat# MAB360, RRID:AB_11212597), and rabbit anti-HCAR1 antibodies diluted in a Tris–HCl buffer (pH 7.8) containing 8.4 mM sodium phosphate, 3.5 mM potassium phosphate, 120 mM sodium chloride, and 1% bovine serum albumin. Sections were incubated in primary antibodies overnight at 22 °C, and subsequently incubated for 2 h at 16 °C with Cy3–Cy5-labeled secondary antibodies (1:200; Jackson ImmunoResearch Labs, Cat# 115-225-146, RRID:AB_2307343, Cat# 115-177-003, RRID:AB_2338719). The samples were counterstained with the DNA stain, TOPRO-3 (1:1000; Thermo Fisher; Cat# T3605). The slices were analyzed by confocal laser microscopy (Carl Zeiss, LSM700, Jena, Germany). A total of three mice were used.

### FACS purification of hypothalamic POMC-EGFP neurons

Ad libitum fed control and Pomc-EGFP mice were sacrificed by cervical dislocation. Tissue from the medial basal hypothalamus region was micro-dissected over a cold surface and keep tissue on cold HBSS. The tissue was then digested with a mix papain/trypsin enzyme (50 U/mL/0.12%, Sigma, USA) for 10 min at 37 °C, followed by mechanical disaggregation using a p1000 plastic tip. Posteriorly, the cellular suspension was incubated for 5 min at 37 °C and mechanically disaggregated using a p200 plastic tip. The cell suspension was then filtered through a 70 µm cell strainer and centrifuge at 300*g* for 5 min at 4 °C. Cells were then resuspended in DPBS (supplemented with 0.5%BSA) to continue with the FACS. Fluorescence-activating cell sorting was performed using an influx cell sorter (FACS Aria-III, BD Biosciences, San Jose, CA, USA), and GFP signals were detected utilizing the FITC detector. The hierarchical cell gating was set according to cell size (FSC) vs cell granularity (SSC), followed by doubles removal. Positive events were considered from a FITC intensity greater than 10^3^ (based on WT control), sorting was performed on purity mode into an Eppendorf tube containing lysis buffer from RNeasy micro kit (Qiagen, Hilden, Germany). For conventional PCR, total RNA extraction and cDNA synthesis was performed using the iScript™ cDNA synthesis kit (BioRad, Hercules, CA, USA). Parallel reactions were performed in the absence of reverse transcriptase to control for the presence of contaminant DNA. For amplification, a cDNA aliquot in a volume of 12.5 mL containing 20 mM Tris–HCl (pH 8.4), 50 mM KCl, 1.6 mM MgCl_2_, 0.4 mM dNTPs, 0.04 units of Taq DNA polymerase (Gibco-BRL, Carlsbad, CA, USA), and 0.4 mM primers was incubated at 95 °C for 4 min, followed by 40 cycles at 95 °C for 15 s, 55 °C for 30 s and 72 °C for 5 min and a final extension of 7 min at 72 °C. PCR products were separated by 1.2% agarose gel electrophoresis and visualized by staining with ethidium bromide^62^.

### Statistical analysis and image processing

All the analyzed values were calculated as the average over each determination. For electrophysiological data, significant differences were determined using the non-parametric Mann–Whitney test. Electrophysiological data were analyzed using GraphPad Prism 5.0 software (GraphPad Software Inc., San Diego, CA, USA: www.graphpad.com). Digital images were analyzed using ImageJ image analysis software (National Institutes of Health, Bethesda, MD: www.rsb.info.nih.gov/ij/).

## Supplementary Information

Supplementary Information.

## Data Availability

All original data will be made available upon reasonable request.
