# Supplementary material for: Lactate activates hypothalamic POMC neurons by intercellular signaling
Source: Sci Rep. 2021 Nov 4;11:21644. doi: 10.1038/s41598-021-00947-7 (PMC8569171; doi:10.1038/s41598-021-00947-7)
Supplement: Supplementary file 1 — Supplementary Information. [file 41598_2021_947_MOESM1_ESM.pdf]

Lactate activates hypothalamic POMC neurons by intercellular signaling

Órdenes P[1], Villar PS[2], Tarifeño-Saldivia E[3], Salgado M[1], Elizondo-Vega R[1], Araneda RC[2*], García-Robles MA[1*]

[1]Laboratorio de Biología Celular, Departamento de Biología Celular, Facultad de Ciencias Biológicas, Universidad de Concepción

[2]Department of Biology, University of Maryland, College Park, Maryland 20742

[3]Gene Expression and Regulation Laboratory, Departamento de Bioquímica y Biología Molecular, Facultad de Ciencias Biológicas, Universidad de Concepción.

Corresponding authors: María A. García-Robles, PhD, Laboratorio de Biología Celular, Departamento de Biología Celular Facultad de Ciencias Biológicas Universidad de Concepción Concepción, Chile

Email: mgarcia@udec.cl and

Ricardo C. Araneda, PhD, Department of Biology, Bioscience Research Bldg. R-1114 University of Maryland College Park, MD 20742 USA

raraneda@umd.edu

Supplementary Methods

Immunoblotting

Total protein extracts were obtained from murine cerebellum, hippocampus, cortex and basal hypothalamus samples homogenized with the following solution: 0.3 mM sucrose, 3 mM DTT, 1 mM EDTA, 2 mg/mL pepstatin A, 100 mg/mL PMSF, 2 mg/mL leupeptin, and 2 mg/mL aprotinin. The basal hypothalamus was dissected from fresh brains over an ice-cold surface by making two transverse cuts, one at the optic chiasm and another just before the mammillary bodies, dissecting the area closest to the 3V [50]. Subsequently, the samples were sonicated on ice at 300 W (Sonics & Material INC., VCF1, Newtown, CT, US), three times, for 10 s. After centrifugation at 8000 g for 10 min, proteins were resolved by SDS PAGE (50 mg/lane), in a 10% (w/v) polyacrylamide gel, and transferred to PVDF membranes (0.45 mm pore, Amersham Pharmacia Biotech., Piscataway, NJ), and probed with rabbit anti-HCA1 (1:1000, Sigma-Aldrich Cat# SAB1300089, RRID:AB_10603645) and anti-β actin (1:10000, Santa Cruz Biotechnology Cat# sc-47778 HRP, RRID:AB_2714189) antibodies. After extensive washing of the primary antibodies, the PVDF membranes were incubated for 2 h., at 4 °C, with peroxidase-labeled anti-rabbit or anti-mouse IgG (1:5000; Jackson ImmunoResearch Labs Cat# 711-035-152, RRID:AB 10015282). The reaction was developed using the Western Lightening Plus-ECL kit (Perkin Elmer; Cat# NEL103001EA) and imaged with a luminescent chemo and fluorescence imaging equipment (PXi, Syngene).

Figures

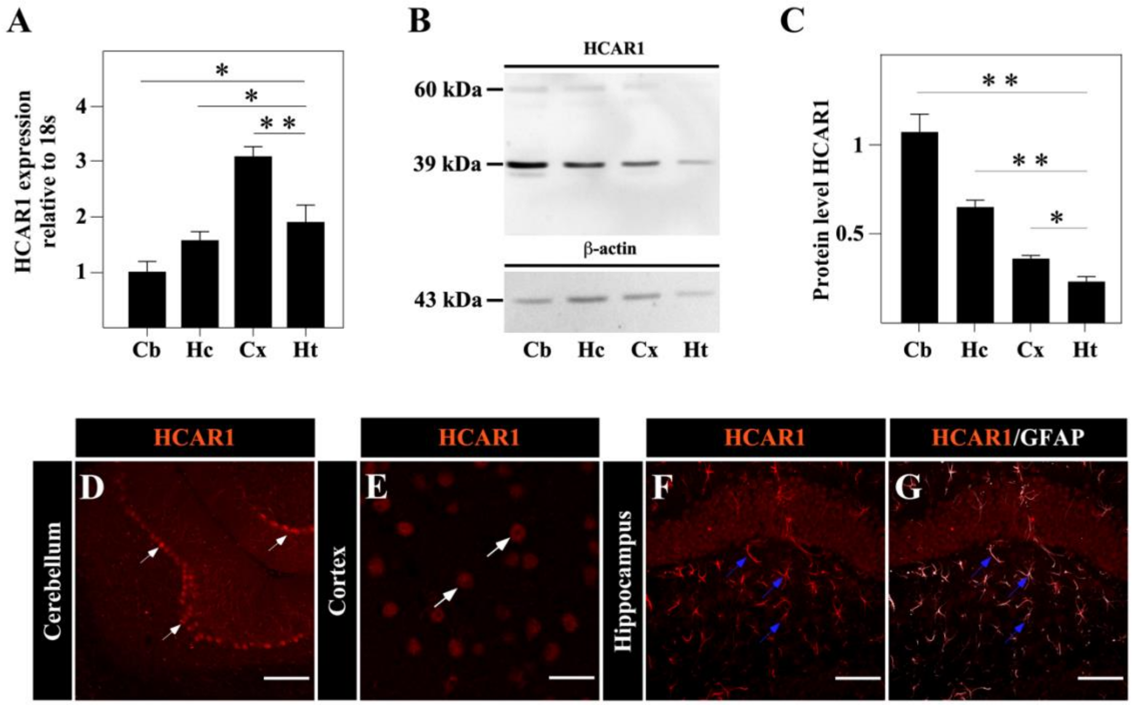

Supplementary Figure S1. Brain HCAR1 expression and localization (A) The HCAR1 sequence was amplified by qRT PCR using specific primers to HCAR1 and 18S as a housekeeping control gene. Data was normalized to the number of copies found in the cerebellum. Amplified PCR products for the cerebellum, hippocampus, cortex, and hypothalamus. (B-C) HCAR1 western blot in total protein extract of cerebellum, hippocampus, cortex, and hypothalamus and quantification relative to β-actin. * p<0.05, **p<0.01. (D-G) Immunolocalization of HCAR1 (red) and GFAP (white). HCAR1 was detected in Purkinge neurons (D, arrows), cortical neurons (E, arrows) in hippocampal astrocytes (F-G, blue arrows). Scale bar (D, F, G); 100 μm, E; 30 μm.

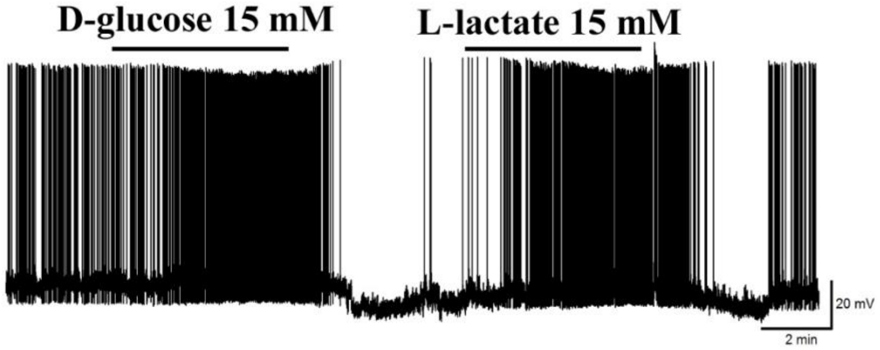

Supplementary Figure S2. Glucose and lactate excite POMC neurons in POMC-EGFP mice model. Current clamp recording in a representative POMC-EGFP neuron. This neuron is excited by D-glucose (15 mM) and L-lactate (15 mM). During the basal and recovery conditions the slice was perfused with aCSF containing 1mM glucose. The resting membrane potential of this neuron was –55 mV.

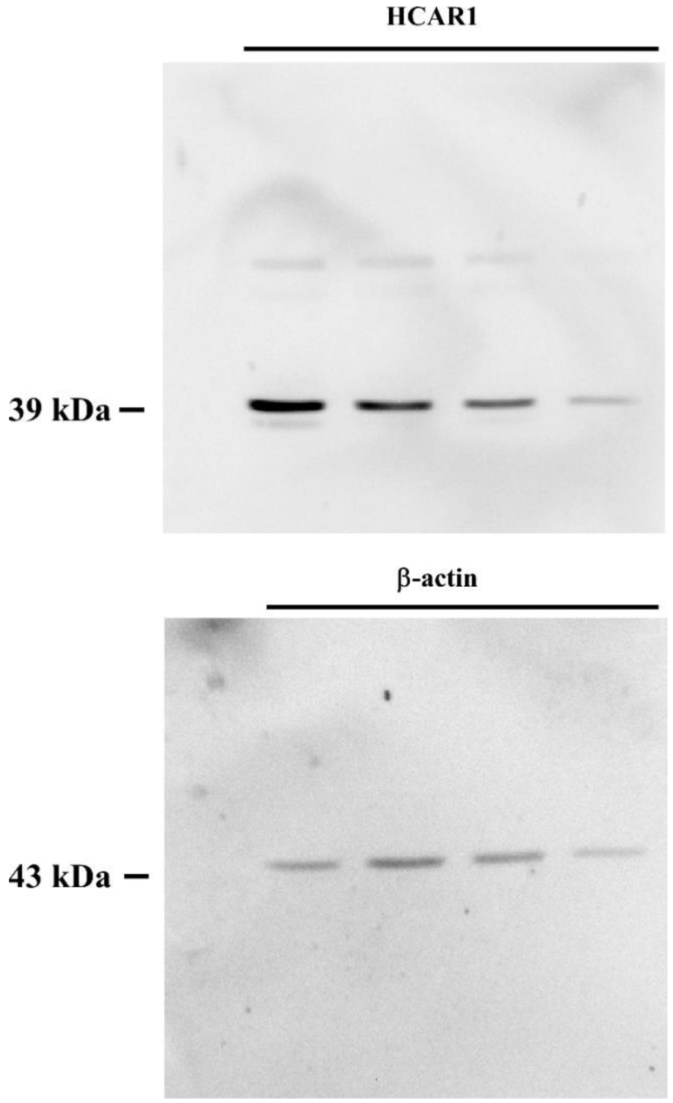

Figure supplementary S3. Uncropped version of the western blots presented in the Supplementary Figure S1.

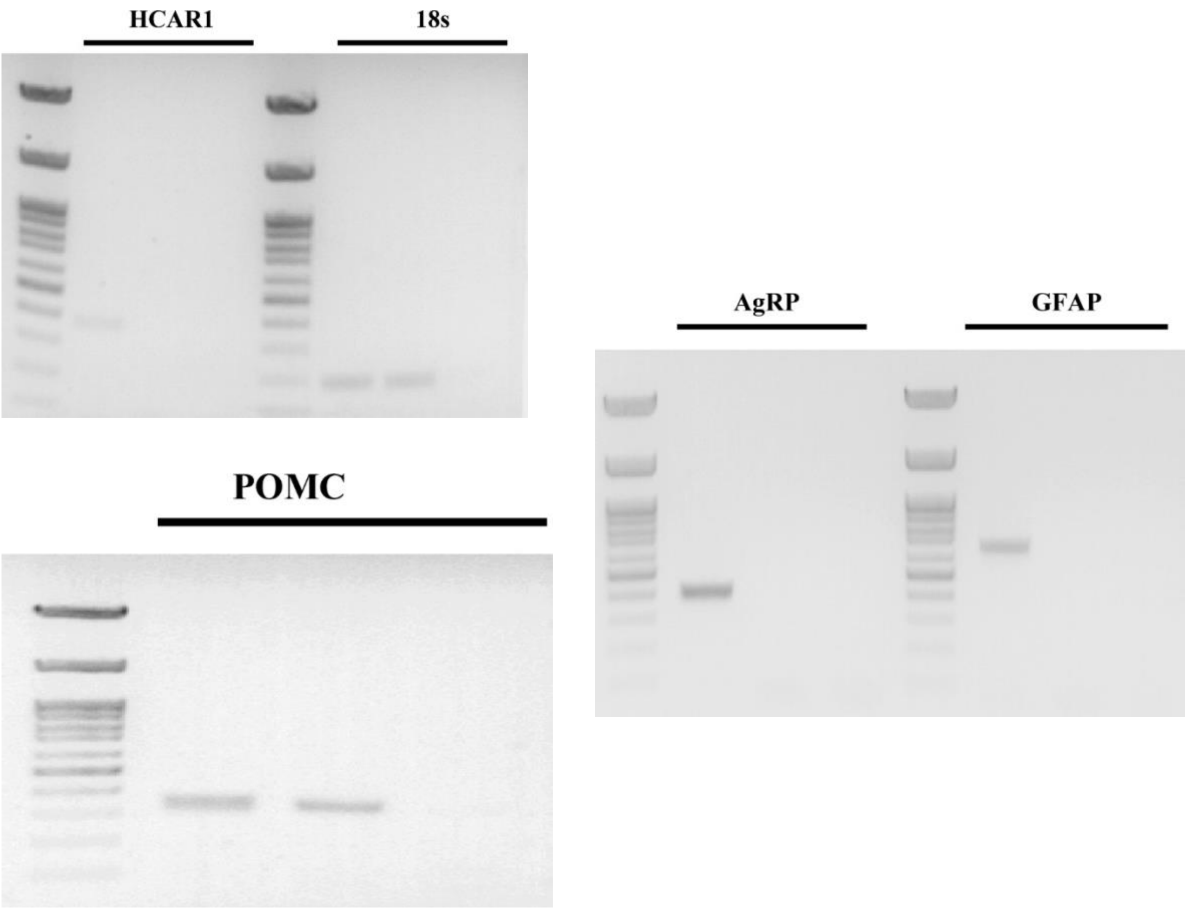

Supplementary Figure S4. Uncropped version of the PCR presented in the figure 5D.
